# Utility of standing office blood pressure in detecting hypertension in healthy adults

**DOI:** 10.1038/s41598-023-42297-6

**Published:** 2023-09-20

**Authors:** John M. Giacona, Weerapat Kositanurit, Jijia Wang, Ursa B. Petric, Ghazi Khan, Danielle Pittman, Jon W. Williamson, Scott A. Smith, Wanpen Vongpatanasin

**Affiliations:** 1 Hypertension Section, Department of Internal Medicine, University of Texas Southwestern Medical Center, Dallas, USA; 2 Department of Applied Clinical Research, School of Health Professions, University of Texas Southwestern Medical Center, Dallas, USA; 3 Department of Physiology, Faculty of Medicine, Chulalongkorn University, Bangkok, Thailand; 4 Cardiology Division, Department of Internal Medicine, University of Texas Southwestern Medical Center, 5323 Harry Hines Blvd., H4.130, Dallas, TX 75390 USA

**Keywords:** Cardiology, Hypertension

## Abstract

Current guidelines recommend office blood pressures (BP) be taken in a seated position when screening for hypertension (HTN). Seated BP is known to have limited accuracy in detecting high BP, while the utility of standing BP in diagnosing HTN is unknown. We conducted a cross-sectional study to determine the incremental value of standing BP in diagnosing HTN. Seated, standing, and 24-h ambulatory BPs (ABPM) were obtained in adults without known cardiovascular disease, HTN, or BP medication use. Presence of HTN was defined by the 2017 ACC/AHA and the 2023 ESH HTN guidelines based on ABPM. Area under the receiver-operating-characteristic curve (AUROC) was used to evaluate the diagnostic accuracy of seated and standing BP. Sensitivity and specificity of standing BP was determined using cut-offs derived from Youden’s Index, while sensitivity and specificity of seated BP was determined using the cut-off of 130/80 mmHg and by 140/90 mmHg. Among 125 participants (mean age 49 ± 17 years; 62% female; 24% Black), 33.6% of them had HTN. Sensitivity and specificity of seated systolic BP (SBP) was 43% and 92%, respectively. Cut-offs selected by Youden’s index for standing SBP/diastolic BP (DBP) were 124/81 mmHg according to the 2017 ACC/AHA HTN guidelines, and 123.5/83.5 mmHg according to the 2023 ESH HTN guidelines. Sensitivity and specificity of standing SBP was 71% and 67%, respectively. The AUROC of standing SBP (0.81 [0.71–0.92]) was significantly higher than seated SBP (0.70 [0.49–0.91]), when HTN was defined as average 24-h SBP ≥ 125 mmHg. Moreover, the addition of standing to seated SBP (0.80 [0.68–0.92]) improved HTN detection when compared to seated SBP. These patterns were consistent for both the 2017 ACC/AHA and the 2023 ESH definitions for HTN. In summary, standing BP, alone or in combination with seated BP, outperformed seated BP alone in diagnosing HTN in adults.

## Introduction

Hypertension (HTN) is the main risk factor for cardiovascular disease (CVD) and effects approximately 46% of the U.S. population^1–3^. Office blood pressure (BP) measurement has been the most used method to characterize a patient’s BP load for many decades^1,4,5^. At present, all practice guidelines and consensus statements have recommended patients have office BP measured in a seated position when being screened for HTN and mention very little regarding taking BP in other positions such as supine or standing^1,4,6^. However, a recent meta-analysis investigating the accuracy of screening for HTN in U.S. adults revealed that the pooled sensitivity of office BP was only 54% with specificity of 90%, when taken in a single visit^7^. The low sensitivity of seated office BP measurements can result in misdiagnosis or delays in treatment, which may pose increased risk of cardiovascular complications. The limited accuracy of office BP in diagnosing HTN and predicting cardiovascular risk has led many guidelines to recommend out-of-office BP measurement, including home BP and 24-h ambulatory BP (ABPM) as diagnostic tools^1,6^. The advantage of 24-h BP monitoring over home BP monitoring is ability to capture BP load throughout the daytime and nighttime.

However, previous studies demonstrate that orthostatic hypotension (OH) and orthostatic hypertension (OHT) are associated with cardiovascular morbidity and mortality^8–15^. Moreover, the prevalence of OHT has been reported to be 1.2–2.6% in young-to-middle-aged adults, and the presence of OHT carries increased risk for development of overt HTN, increased risk of cardiovascular disease, and increased all-cause mortality^8,9,16–20^. Despite the increasing investigations into orthostatic BP, previous studies have not incorporated standing BP in the diagnosis of HTN. Accordingly, we performed an investigation in a group of adults free of cardiovascular disease and without anti-HTN medication use to determine if the addition of standing office BP to seated office BP would increase diagnostic accuracy for the presence of HTN.

## Methods

### Study design and population

**Figure 1:**
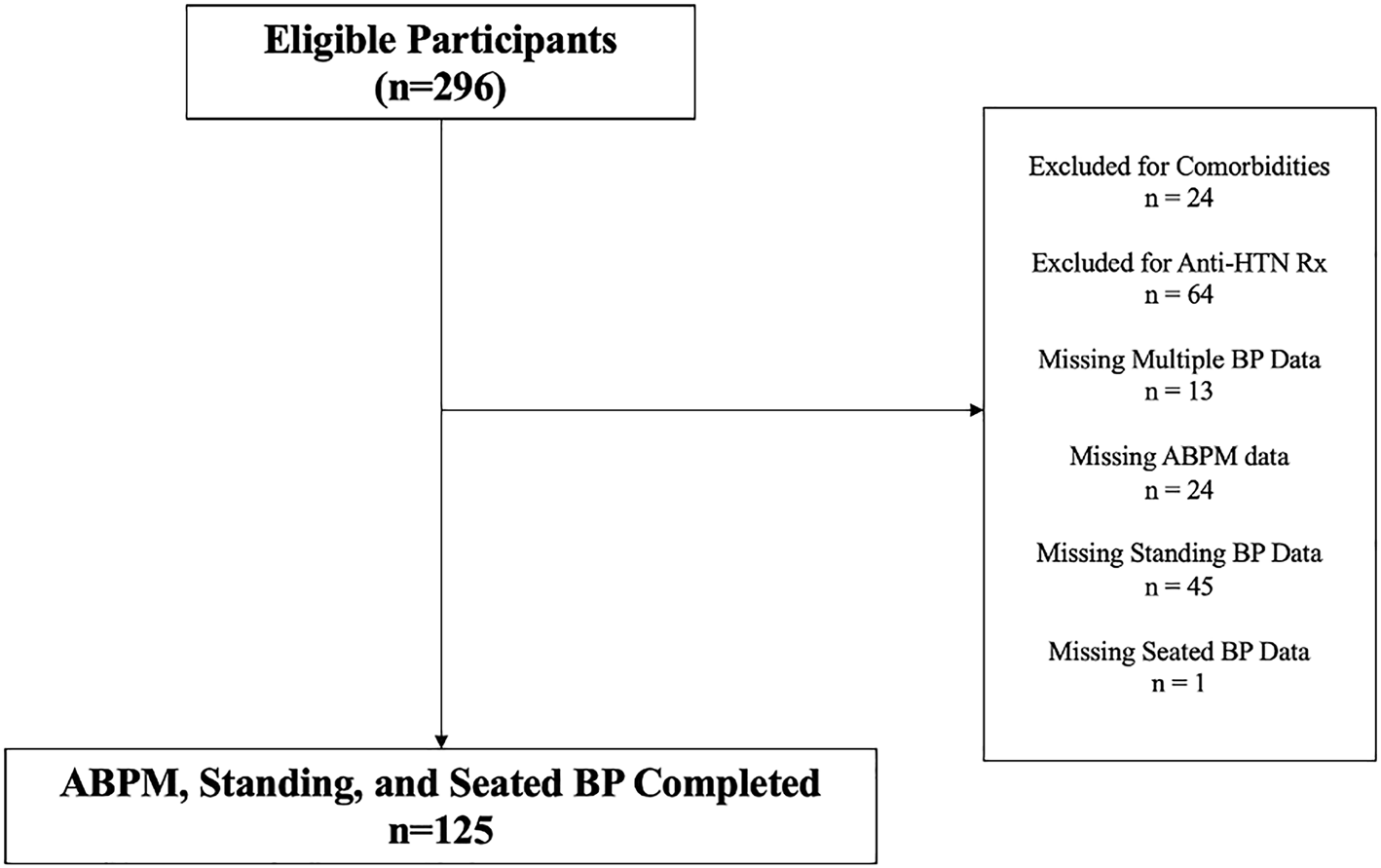
Disposition of study participants. Consolidated standards of reporting trials of the participants included in the study. *ABPM* ambulatory blood pressure monitoring, *Anti-HTN Rx* anti-hypertensive medications, *BP* blood pressure.

All studies performed were approved by the Institutional Review Board at the University of Texas Southwestern Medical Center, all studies performed were in accordance with current guidelines/regulations, as appropriate, and written informed consent was obtained from all subjects. We retrospectively obtained participant data from the pool of subjects who have participated in clinical trials conducted in our hypertension research center. Studies were conducted in participants aged 18–80 years without previous history of HTN, cardiopulmonary disease, chronic kidney disease, use of anti-HTN medications, estimated glomerular filtration rate (eGFR) < 60 mL/min/1.73 m^2^, diabetes mellitus, pregnancy, history of substance abuse, or current smoker. As shown in Fig. 1, there were 296 total number of eligible participants screened, of which 83 were excluded for missing or inadequate blood pressure data, 64 were excluded for being on anti-HTN medications, and 24 were excluded due to presence of comorbidities, leaving 125 to be included in final analyses.

### Experimental measures

Self-reported variables included sex, race, and ethnicity. Body mass index (BMI), serum creatinine, fasting plasma glucose, eGFR, 24-h ABPM, seated and standing office BP were collected by research staff. Participants underwent a medical history and physical exam, and had BP measured in both seated and standing positions that was followed by obtaining a 24-h ABPM.

#### 24-hour ambulatory blood pressure monitoring (ABPM)

ABPM was conducted using validated Spacelabs model 90207 or 90227 monitors (Spacelabs, Snoqualmie, WA)^21–23^. Participants were fitted with the appropriate size BP cuff on the non-dominant arm. Monitors were initialized to define daytime as 0600–2159 h and nighttime as 2200–0559 h. Measurements were obtained every 20 min during the day and every 30 min at night. Duration of ambulatory monitoring was 24 h, and only patients with the minimum recommended number of ≥ 20 readings during the daytime period, and ≥ 7 readings during the nighttime period, and those with > 70% successful measurements, were considered acceptable, according to guidelines^1,6^.

#### Seated and standing office BP

Office BP was measured using a Welch Allyn Vital Signs 6000 series (model 901060, Welch Allyn, Skaneateles Falls) oscillometric BP monitor. Participants were fitted with an appropriate size BP cuff on the non-dominant arm (bladder length: 75–100% of the upper arm circumference, and bladder width: 37–50% of the upper arm circumference). All participants then had seated office BP taken after resting quietly for 5 min, with arm supported on a table at the level of the heart, back supported, both feet resting flat on the ground (uncrossed) and were instructed to stay silent during 5-min rest period prior to BP measurement, and during all sequential BP measurements, according to the current practice guidelines^1^. Participants had been asked to refrain from caffeine, exercise, and smoking for at least 30 min before measurements. Seated measurements were performed 3 times sequentially (separated by 1 min). Standing office BP was measured immediately after seated office BP measurements had been taken, with participants arm supported on a table at heart level and measurements performed 3 times sequentially (separated by 1 min). The second and third readings of both seated and standing BP were averaged for analyses.

### Statistical analyses

Statistical analyses were performed using SAS version 9.4. Participant characteristics are reported as mean ± standard deviation (SD) for continuous data and number (%, percentage) for categorical data. Office BP data had coefficient of variation additionally reported. Categorical variables were compared using the Fisher exact test or Chi-square when appropriated, and continuous variables were compared using two-sample T-test after checking normality. Predictors of standing and seated office BP were determined by multivariable linear regression analyses. All p-values were 2-tailed. A p-value < 0.05 was considered statistically significant. It was determined that a sample size of n = 122 would provide at least 80% power to detect a minimum sensitivity of 0.7 estimated from setting prevalence of HTN at 40% and 0.5 for the sensitivity of the null hypothesis^24^. Participants were grouped by presence or absence of HTN as determined by the 2017 ACC/AHA HTN guidelines for average 24-h ABPM ≥ 125/75 mmHg, and by the 2023 ESH HTN guidelines for average 24-h ABPM ≥ 130/80 mmHg. These criteria were also used for calculating sensitivity and specificity of diagnostic tests as true HTN status. Validity measures of sensitivity, specificity, positive predictive value (PPV), and negative predictive value (NPV) were calculated with their corresponding 95% confidence intervals (CI). McNemar’s test was performed to assess the significance of the difference between sensitivity and specificity of standing and seated office BP for determining the presence of HTN.

Overall diagnostic accuracy of seated and standing BP in diagnosing HTN was assessed using area under the receiver-operating-characteristic curve (AUROC)^25^. The presence of HTN when determining the AUROC(s) was treated as binary under several established cut-offs termed “case definition” in the present study. The case definitions were defined as 24-h SBP ≥ 125 mmHg, 24-h DBP ≥ 75 mmHg, Daytime SBP ≥ 130 mmHg, Daytime DBP ≥ 80 mmHg, according to 2017 ACC/AHA guidelines, and 24-h SBP ≥ 130 mmHg, 24-h DBP ≥ 80 mmHg, Daytime SBP ≥ 135 mmHg, and Daytime DBP ≥ 85 mmHg, according to 2023 ESH guidelines^1,6^. Each case definition was used to sort participants into “case” and “control” groups based off each respective definition. These groups determined true presence of HTN, then the AUROC of seated and standing BPs was determined. Bayes factor (BF) was calculated on 5000 bootstrap samples and used to assess the significance of the difference between AUROC of seated and standing BP. Based on bootstrap samples, the number of times model A (i.e., seated BP) outperforms model B (i.e., standing BP) was divided by the number of times model B outperforms model A, to form the Bayes factor^26^. A Bayes factor of 3 or greater was considered a significant cutoff^27,28^. Sensitivity and specificity of seated BP was determined using the cut-offs of 130/80 mmHg, and 140/90 mmHg, according to most recent 2017 ACC/AHA, and 2023 ESH guidelines, respectively. There are no established cut-offs for diagnosing hypertension from standing BP. Therefore, we employed a statistical method known as Youden's Index, in conjunction with ABPM cut-offs (based on the 2017 ACC/AHA guidelines or the 2023 ESH guidelines), to identify the optimal cut-off points for standing SBP and DBP where the balance between sensitivity and specificity is maximized^1,29^.

## Results

### Participant characteristics

The participant characteristics of our cohort (n = 125) are shown in Table 1. Prevalence of HTN based on average 24-h ABPM was 33.6% (n = 42). Among those found to have HTN on 24-h ABPM, the mean age was 55.7 ± 14.5 years, with 57% female, 55% White adults, 29% Black/African American adults, and 16% Asian adults. There were 16% Hispanic/Latino (of those, 100% self-identified as White adults) in the HTN group. Those found to be normotensive on average 24-h ABPM were younger with a mean age of 45.3 ± 17.2 years and had similar presence of females (64%), those self-identifying as White adults (64%), Black/African American adults (22%), and Asian adults (12%). The normotensive group also had similar proportion of Hispanic/Latino of 16% (of those, 85% self-identified as White adults, and 15% self-identified as Black/African American adults). Similarly, BMI, serum creatinine, fasting plasma glucose, and eGFR were not significantly different between the groups. In a multivariable linear regression analysis, age was a positive significant predictor of seated office systolic BP, while female sex was a significant negative predictor for standing office systolic BP, after adjusting for BMI, fasting plasma glucose, eGFR, race and ethnicity (Supplemental Table 1). Coefficient of variation for office BP are shown in Supplemental Table 2. Proportion of those with normotension, seated and standing HTN, and seated or standing HTN are shown in Supplemental Fig. 1.

**Table 1:** Characteristics of participants.

Variables	Cohort (n = 125)	Hypertensive (n = 42)	Normotensive (n = 83)	*P*-value
Age (years), mean ± SD	49.0 ± 17.0	55.7 ± 14.5	45.3 ± 17.2	0.001
Female, n (%)	77 (62%)	24 (57%)	53 (64%)	0.56
Race, n (%)				
White	76 (61%)	23 (55%)	53 (64%)	0.51
Black/African American	30 (24%)	12 (29%)	18 (22%)	–
Asian	17 (14%)	7 (16%)	10 (12%)	–
Ethnicity, n (%)				
Hispanic/Latino	18 (14%)	5 (16%)	13 (16%)	0.78
BMI (kg/m^2^), mean ± SD		27.8 ± 4.2	26.6 ± 5.6	0.15
Serum creatinine (mg/dL), mean ± SD		0.84 ± 0.17	0.82 ± 0.16	0.43
Fasting glucose (mg/dL), mean ± SD		95.2 ± 12.5	92.5 ± 12.9	0.32
eGFR (mL/min/1.73m^2^), mean ± SD		91.3 ± 19.2	99.6 ± 19.4	0.05
Office BP (mmHg), mean ± SD				
Seated systolic BP		134 ± 20	120 ± 9	< 0.001
Seated diastolic BP		80 ± 11	75 ± 5	0.002
Standing systolic BP		136 ± 24	121 ± 9	< 0.001
Standing diastolic BP		85 ± 13	78 ± 6	< 0.001
Office HR (bpm), mean ± SD				
Sitting HR		71 ± 11	71 ± 11	0.82
Standing HR		80 ± 13	78 ± 12	0.43
Ambulatory BP (mmHg), mean ± SD				
24-h SBP, mean ± SD		130 ± 12	111 ± 7	< 0.001
24-h DBP, mean ± SD		78 ± 7	68 ± 4	< 0.001
Daytime SBP, mean ± SD		134 ± 13	115 ± 7	0.002
Daytime DBP, mean ± SD		81 ± 8	72 ± 5	< 0.001
Nighttime SBP, mean ± SD		121 ± 14	102 ± 13	< 0.001
Nighttime DBP, mean ± SD		70 ± 7	61 ± 5	< 0.001
Ambulatory HR (bmp), mean ± SD				
24-h HR		75 ± 9	70 ± 9	0.002
Daytime HR		77 ± 9	72 ± 9	0.004
Nighttime HR		71 ± 10	64 ± 10	0.001

Categorical data are presented as number and percentage and continuous data are presented as mean and standard deviation. *P* < 0.05 was considered statistically significant.

*BMI* body mass index, *BP* blood pressure, *eGFR* estimated glomerular filtration rate, *HR* heart rate, *SD* standard deviation.

### AUROC comparing seated and standing office blood pressure

**Figure 2:**
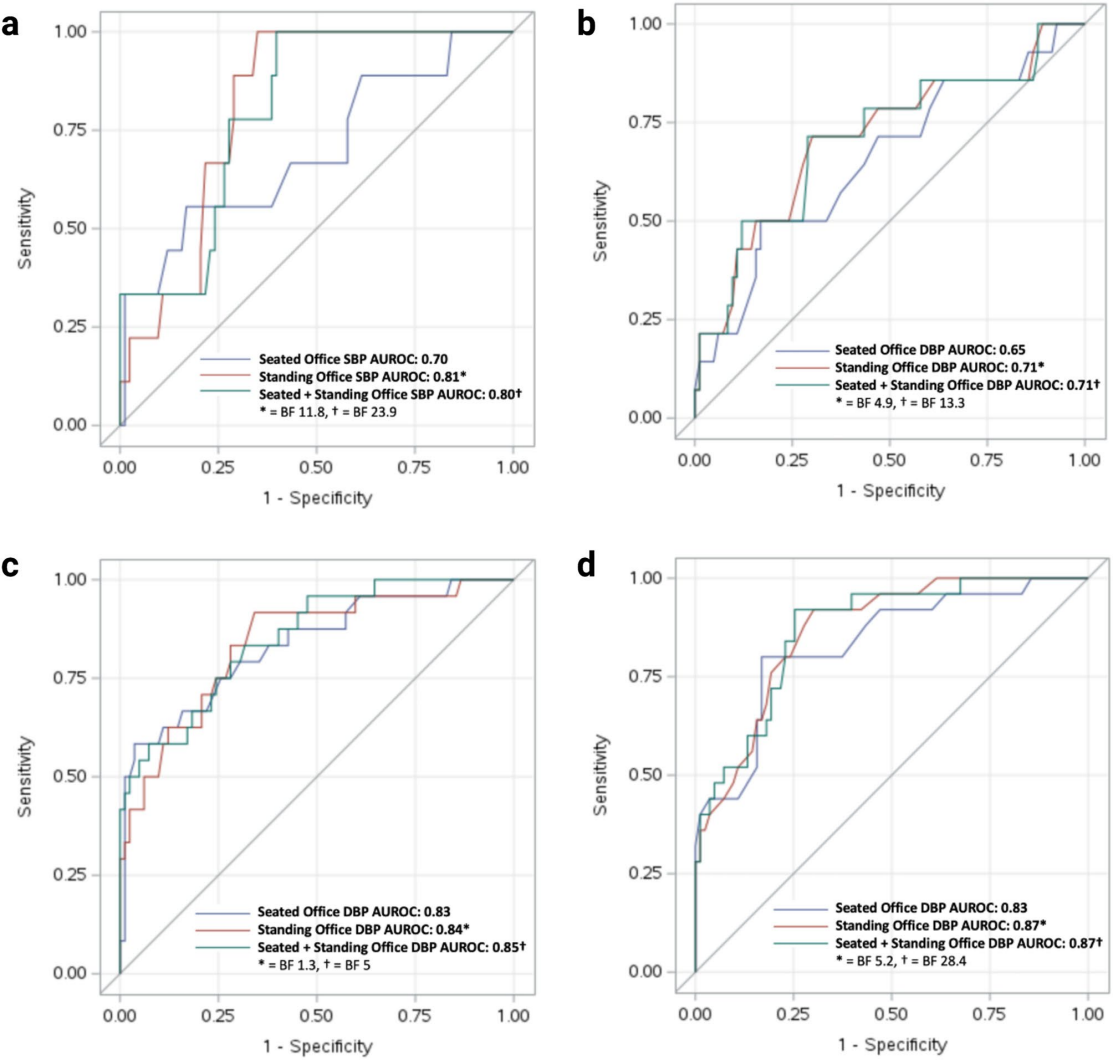
AUROCs of SBP and DBP. AUROCs of (**a**) seated vs. standing SBP, and of (**b**) seated vs. standing DBP, as defined by reference standard of average 24-h SBP of  ≥ 125 mmHg and 24-h DBP of  ≥ 75 mmHg, respectively, and AUROCs of (**c**) seated vs. standing SBP, and (**d**) seated vs. standing DBP, as defined by reference standard of Daytime SBP of  ≥ 130 mmHg and DBP of  ≥ 80 mmHg, respectively, to detect presence of hypertension. *AUROC* area under the receiver-operating-characteristic curve, *BF* Bayes factor, *DBP* diastolic blood pressure, *SBP* systolic blood pressure.

The AUROC for standing office SBP (0.81 [0.71–0.92]) was significantly higher than the AUROC for seated office SBP (0.70 [0.49–0.91]) in diagnosing HTN when case status was defined as having an average 24-h SBP ≥ 125 mmHg (BF = 11.8, Table 2: 2017 ACC/AHA Guideline for HTN, and Fig. 2a). Likewise, the AUROC for standing office DBP (0.71 [0.55–0.88]) was significantly higher than the AUROC for seated office DBP (0.65 [0.49–0.82]) in diagnosing HTN when case status was defined as having an average 24-h DBP ≥ 75 mmHg (BF = 4.9, Table 2: 2017 ACC/AHA Guideline for HTN, and Fig. 2b). The AUROC for standing office DBP (0.87 [0.80–0.94]) was significantly higher than the AUROC for seated office DBP (0.83 [0.74–0.93]) in diagnosing HTN when case status was defined as having daytime DBP ≥ 80 mmHg (BF = 5.2, Table 2: 2017 ACC/AHA Guideline for HTN). Furthermore, the AUROCs for the addition of standing office BP to seated office BP improved the accuracy of detecting HTN compared to seated office BP alone when case status was defined as having an average 24-h SBP/DBP ≥ 125/75 mmHg or daytime SBP/DBP ≥ 130/80 mmHg (all BFs > 3, Table 2 and Fig. 2). Conversely, the AUROCs for standing office SBP (0.84 [0.75–93]) and seated office SBP (0.83 [0.73–0.93]) were not significantly different when case status was defined as a daytime SBP ≥ 130 mmHg (BF = 1.3, Table 2). Moreover, the AUROCs for the addition of standing office BP to seated office BP improved the accuracy of detecting HTN compared to seated office BP alone when case status was defined as having an average 24-h SBP/DBP ≥ 130/80 mmHg or daytime SBP/DBP ≥ 135/85 mmHg (all BFs > 3, Table 2: 2023 ESH Guideline for HTN).

**Table 2:** Bayes factor comparing AUROC for seated and standing blood pressures.

Case definition	AUROC	Bayes factor
2017 ACC/AHA guideline for hypertension		
Average 24-h SBP ≥ 125 mmHg	Seated: 0.70 [0.49–0.91] Standing: 0.81 [0.71–0.92] Combination: 0.80 [0.68–0.92]	11.8* 23.9*
Average 24-h DBP ≥ 75 mmHg	Seated: 0.65 [0.49–0.82] Standing: 0.71 [0.55–0.88] Combination: 0.71 [0.55–0.88]	4.9* 13.3*
Daytime SBP ≥ 130 mmHg	Seated: 0.83 [0.73–0.93] Standing: 0.84 [0.75–0.93] Combination: 0.85 [0.77–0.94]	1.3 5.0*
Daytime DBP ≥ 80 mmHg	Seated: 0.83 [0.74–0.93] Standing: 0.87 [0.80–0.94] Combination: 0.87 [0.79–0.94]	5.2* 28.4*
2023 ESH guideline for hypertension		
Average 24-h SBP ≥ 130 mmHg	Seated: 0.77 [0.64–0.90] Standing: 0.80 [0.68–0.92] Combination: 0.80 [0.69–0.91]	2.2 5.9*
Average 24-h DBP ≥ 80 mmHg	Seated: 0.87 [0.79–0.96] Standing: 0.89 [0.83–0.96] Combination: 0.89 [0.83–0.96]	2.4 6.5*
Daytime SBP ≥ 135 mmHg	Seated: 0.78 [0.64–0.92] Standing: 0.81 [0.68–0.93] Combination: 0.82 [0.70–0.93]	2.3 8.8*
Daytime DBP ≥ 85 mmHg	Seated: 0.88 [0.79–0.98] Standing: 0.91 [0.86–0.97] Combination: 0.91 [0.84–0.97]	2.6 8.3*

*Statistically significant difference when compared to seated BP (BF > 3).

*ACC* American College of Cardiology, *ABPM* ambulatory blood pressure monitoring, *AHA* American Heart Association, *AUROC curve* area under the receiver-operating-characteristic curve, *BF* Bayes factor, *BP* blood pressure, *ESH* European Society of Hypertension, *SBP* systolic blood pressure.

### Measures of validity comparing seated and standing office blood pressure

To determine sensitivity, specificity, and predictive values of standing office BP, optimal cutoffs used were derived from Youden’s index from both average 24-h SBP/DBP ≥ 125/75 mmHg and daytime SBP/DBP ≥ 130/80 mmHg and found to be 123.5/80.5 and 123.5/81 mmHg, respectively. Therefore, when determining sensitivity and specificity of standing office BP according to the 2017 ACC/AHA HTN guidelines, we utilized the cut-off of 124/81 mmHg. For the 2023 ESH HTN guidelines, optimal cut-offs derived from Youden’s index for 24-h SBP/DBP ≥ 130/80 mmHg and daytime SBP/DBP ≥ 135/85 mmHg were found to be 123.5/83.5 and 133/81.5 mmHg, respectively. Thus, when determining sensitivity and specificity of standing office BP according to the 2023 ESH HTN guidelines, the cut-off of 123.5/83.5 mmHg (which corresponded to the average 24-h ABPM) was utilized. As shown in Table 3, sensitivities were greater for standing office systolic and diastolic BPs when compared to seated office BPs. Conversely, specificities were lower for standing office BPs when compared to seated office BPs. Negative predictive values of standing office systolic and diastolic BPs were higher than that of seated office BPs. While the positive predictive values of standing office systolic and diastolic BPs were lower than seated office BPs. These patterns were consistent for both the 2017 ACC/AHA and the 2023 ESH definitions for hypertension (Table 3).

**Table 3:** Comparison of sensitivity, specificity, negative predictive value, and positive predictive value of seated and standing blood pressures.

	Sensitivity (95% CI)	Specificity (95% CI)	PPV (95% CI)	NPV (95% CI)
2017 ACC/AHA guideline for hypertension				
Seated SBP	43 (28–58)%	92 (86–98)%	72 (54–90)%	76 (68–84)%
Standing SBP^†^	71* (58–85)%	67* (56–76)%	52 (39–65)%	82 (73–91)%
Seated DBP	45 (30–60)%	83 (75–91)%	58 (41–74)%	75 (66–84)%
Standing DBP^†^	67* (52–81)%	70* (71–90)%	53 (40–66)%	81 (71–90)%
2023 ESH guideline for hypertension				
Seated SBP	42 (22–61)%	97 (94–100)%	77 (54–100)%	88 (81–94)%
Standing SBP^‡^	83* (68–98)%	60* (51–70)%	33 (21–45)%	94 (88–100)%
Seated DBP	29 (11–47)%	99 (97–100)%	88 (65–100)%	85 (79–92)%
Standing DBP^‡^	58* (39–78)%	77* (69–85)%	38 (22–53)%	89 (82–95)%

*Statistically significant difference when compared to seated BP (*P* < 0.05).

^†^Optimal cutoffs derived from Youden’s index for standing BP: 124/81 mmHg (based on 2017 ACC/AHA Guidelines).

^‡^Optimal cutoffs derived from Youden’s index for standing BP: 123.5/83.5 mmHg (based on 2023 ESH Guidelines).

*ACC* American College of Cardiology, *AHA* American Heart Association, *BP* blood pressure, *CI* confidence interval, *DBP* diastolic BP, *ESH* European Society of Hypertension, *NPV* negative predictive value, *PPV* positive predictive value, *SBP* systolic BP.

## Discussion

The new major findings in the present study investigating the utility of standing office BP measurement in detecting hypertension in young-to-middle-aged adults not taking anti-hypertensive medications were twofold. First, the diagnostic threshold for standing BP is consistent regardless of whether the definition for hypertension is based on average 24-h ambulatory BP ≥ 125/75 mmHg, or daytime ambulatory BP ≥ 130/80 mmHg. Second, we found that the addition of standing office BPs to seated office BPs enhances the diagnostic accuracy in detecting hypertension when compared to traditional seated office BPs alone and this finding was consistent for both the 2017 ACC/AHA and 2023 ESH definitions for hypertension, which provides further support for the validity of standing BP.

Results from a recent meta-analysis investigating the accuracy of hypertension screening in U.S. adults demonstrate the current accuracy of using traditional office BP methods during a single visit for the screening of hypertension yields a pooled sensitivity of 54% and a pooled specificity of 90% based on 15 studies included (n = 11,309)^7^. Further, the meta-analysis reported that accuracy on a repeat office visit yielded a higher pooled sensitivity of 80% and a lower pooled specificity of 55% versus the initial visit based on 8 studies included (n = 53,183)^7^. Our study found that on an initial screening visit, standing office SBP yielded a sensitivity of 71%, which is higher than the sensitivity of seated office SBP. Additionally, the AUROC of 0.81 found for standing office SBP further demonstrates excellent diagnostic performance and highlights that standing office BP has acceptable discriminative capabilities in identifying the presence of hypertension in adults. Our results also showed that the combination of seated and standing office BP resulted in greater discriminative capability to detect hypertension when compared to using seated office BP alone regardless of whether the 2017 ACC/AHA or 2023 ESH definitions for hypertension were used. Although the specificity is reduced to 67–70% when using standing BP measurement as a diagnostic tool, the specificity of standing BP in our study remains higher than the specificity reported in the meta-analysis when the second office BP measurement was conducted to confirm findings of the first office BP measurement^7^. Furthermore, the standing BP cut-off of 124/81 mmHg was remarkably similar whether average 24-h ambulatory BP or daytime ambulatory BP was used as the gold standard. Thus, routine standing BP measurement may facilitate diagnosis of hypertension more rapidly without additional office BP measurement to confirm a diagnosis.

In addition to the improved diagnostic value that standing office BP provides, including an orthostatic BP assessment in an office visit can also inform the clinician of a patient’s future cardiovascular risk. Several prior studies have shown an association between increased standing office BP and cardiovascular morbidity and mortality^8–14^. Specifically, a recent analysis evaluating the change in BP to standing found that the presence of an increase in SBP of > 6.5 mmHg with standing was an independent predictor of major adverse cardiovascular events (MACE) in young-middle age healthy humans^30^. Moreover, the group that displayed “hyperreactivity”—the increased SBP to standing, had a higher prevalence of hypertension based on average 24-h ambulatory BP assessment in addition to the near two-fold increase in MACE [hazard ratio of 1.97 (95% CI 1.10–3.52)] based on a 17.2-year follow-up when compared with those with normal response in SBP to standing^30^. Excessive neurohormonal activation and sympathetic overactivity may be responsible for this observation^18,30^. Likewise, orthostatic hypotension, defined as a reduction in SBP of ≥ 20 or a reduction in DBP of ≥ 10 mmHg one minute after standing from a seated position, has also been shown to be associated with increased cardiovascular mortality^15,31–33^. Taken into context, our results highlight that by obtaining standing office BPs, a clinician can also utilize the advantage of increasing diagnostic accuracy for hypertension in addition to the important prognostic information provided.

**Figure 3:**
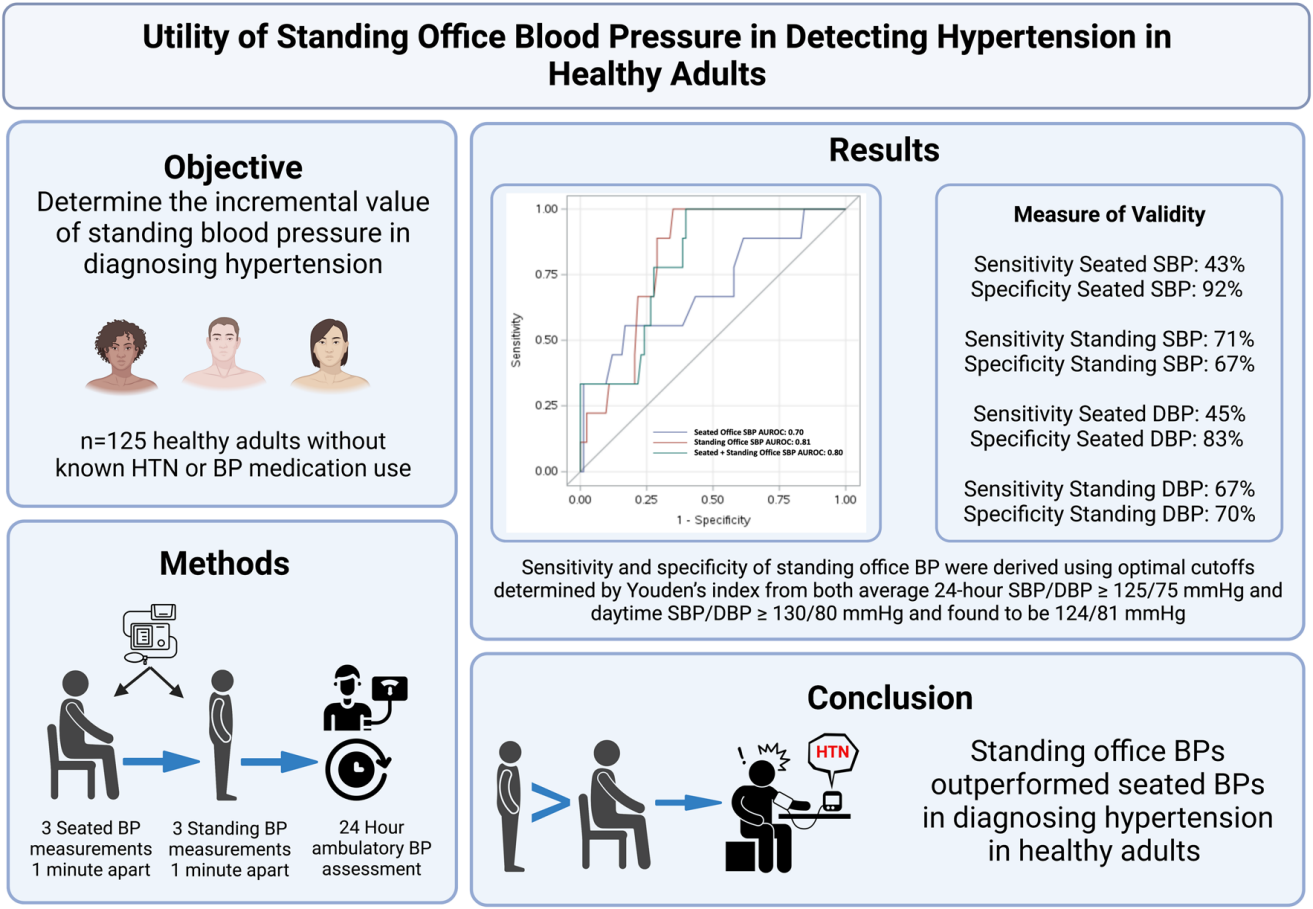
Graphical abstract. Schematic summary of the abstract including main objective, study protocol, essential results, and clinical implications. *AUROC* area under the receiver-operating-characteristic curve, *BP* blood pressure, *DBP* diastolic blood pressure, *HTN* hypertension, *SBP* systolic blood pressure.

Our study is limited in that we only enrolled adults free of comorbidities who were not taking anti-hypertensive medications. Therefore, our results may not be applicable to patients with comorbidities or those on anti-hypertensive medications. Our study is retrospective in design and standing BP measurement was obtained only after seated BP, which does not allow randomization of the order of BP measurement in different positions. Nevertheless, standing BP measurement is almost always performed after seated BP in clinical settings. Despite these limitations, our study had several strengths. Our study protocol and essential findings are shown in Fig. 3. Firstly, we obtained high quality assessment of clinic BP that included 3 BP measurements in the seated position and 3 measurements in the standing position. Secondly, all participants had 24-h ABPM complete within the same 24–48-h period of office BP measurement.

In conclusion, our study is the first to demonstrate that obtaining standing office BP can increase sensitivity during an initial screening visit. Moreover, we show that the combination of both seated office BP and standing office BP performed better than seated office BP alone in detecting hypertension.

## Perspectives

The results of the present study have important clinical implications in the screening and diagnosis of hypertension. Our results suggest that obtaining standing office BPs during an initial screening visit provide a significant advantage through improving diagnostic ability and increase accuracy for detecting hypertension, thereby potentially reducing the need for a second office visit during the screening process. However, further studies are needed to determine the precise role of incorporating standing office BP in the overall diagnostic algorithm for the screening and treatment of hypertension.

### Supplementary Information

Supplementary Information.

## Data Availability

The datasets generated and analyzed during the current study are available from the corresponding author upon reasonable request.
