# Supplementary material for: Utility of standing office blood pressure in detecting hypertension in healthy adults
Source: Sci Rep. 2023 Sep 20;13:15572. doi: 10.1038/s41598-023-42297-6 (PMC10511521; doi:10.1038/s41598-023-42297-6)

SUPPLEMENTAL MATERIAL

Utility of Standing Office Blood Pressure in Detecting Hypertension in Healthy Adults

John M. Giacona, M.P.A.S., PA-C[1,2], Weerapat Kositanurit, M.D.[1,3], Jijia Wang, Ph.D.[2], Ursa B. Petric, B.S.[1], Ghazi Khan, M.D.[1], Danielle Pittman, B.S.N.[1,3], Jon W. Williamson, Ph.D.[2], Scott A. Smith, Ph.D.[2], Wanpen Vongpatanasin, M.D.[1,4]*

[1]Hypertension Section, Department of Internal Medicine, University of Texas Southwestern Medical Center; [2]Department of Applied Clinical Research, School of Health Professions, University of Texas Southwestern Medical Center; [3]Department of Physiology, Faculty of Medicine, Chulalongkorn University, Thailand; [4]Cardiology Division, Department of Internal Medicine, University of Texas Southwestern Medical Center, United States of America

Address correspondence to Dr. Vongpatanasin at the Hypertension Section, Cardiology Division, University of Texas Southwestern Medical Center, 5323 Harry Hines Blvd., H4.130, Dallas, TX 75390. Phone: (214) 648-4403; E-mail: Wanpen.Vongpatanasin @UTsouthwestern.edu

Supplemental Table 1. Determinants of seated and standing systolic BP. Summary of multiple linear regression analyses.

Variable	Coefficient	Standard error	p-value
**Seated Systolic Blood Pressure**			
Age	0.32	0.13	0.01*
Sex (female)	-5.29	2.99	0.08
Race (Black/African American)	-0.20	3.59	0.96
Ethnicity (Hispanic/Latino)	0.89	4.12	0.83
BMI	0.50	0.30	0.10
eGFR	0.01	0.12	0.96
Fasting glucose	0.15	0.12	0.19
**Standing Systolic Blood Pressure**			
Age	0.25	0.14	0.08
Sex (female)	-8.98	3.29	<0.01*
Race (Black/African American)	3.90	3.96	0.33
Ethnicity (Hispanic/Latino)	1.20	4.55	0.79
BMI	0.65	0.33	0.05
eGFR	-0.07	0.12	0.56
Fasting glucose	0.19	0.13	0.14

*$P<0.05$ was considered statistically significant.

BMI, body mass index; eGFR, estimated glomerular filtration rate.

Supplemental Table 2. Seated and standing office BP variability.

Position	Mean BP (mmHg)	SD*	CV†
Seated SBP	124.4	3.2 ± 2.6	0.03 ± 0.04
Seated DBP	76.5	1.9 ± 1.5	0.03 ± 0.02
Standing SBP	125.9	3.2 ± 2.6	0.03 ± 0.02
Standing DBP	80.1	2.0 ± 1.8	0.03 ± 0.02

Within-subject mean BP, SD, and CV were calculated.

* SD presented as mean SD ± SD

† CV presented as mean CV ± SD

CV = (SD of mean BP / mean BP (mmHg))

BP, blood pressure; CV, coefficient of variation; DBP, diastolic blood pressure; SBP, systolic blood pressure; SD, standard deviation.

Supplemental Figure Legends

Supplemental Figure 1 Proportion of Hypertension Diagnosis

Proportions of hypertension based on seated and standing blood pressure. Seated HTN defined by both (a) 2017 ACC/AHA (≥130/80 mmHg), and (b) 2023 ESH (≥140/90 mmHg) guidelines for hypertension. Standing HTN determined by using optimal cutoffs derived from Youden's index using reference ABPM of ≥125/75 mmHg (2017 ACC/AHA) and ≥130/80 mmHg (2023 ESH) which were ≥124/81 mmHg and ≥123.5/83.5 mmHg, respectively.

ACC, American College of Cardiology; AHA, American Heart Association; ESH, European Society of Hypertension; HTN, hypertension.

Supplemental Figure 1.

a

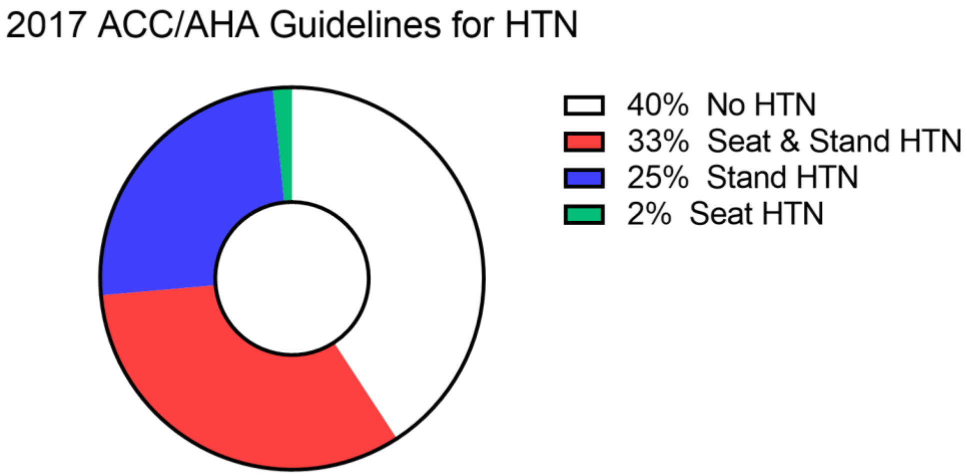

b

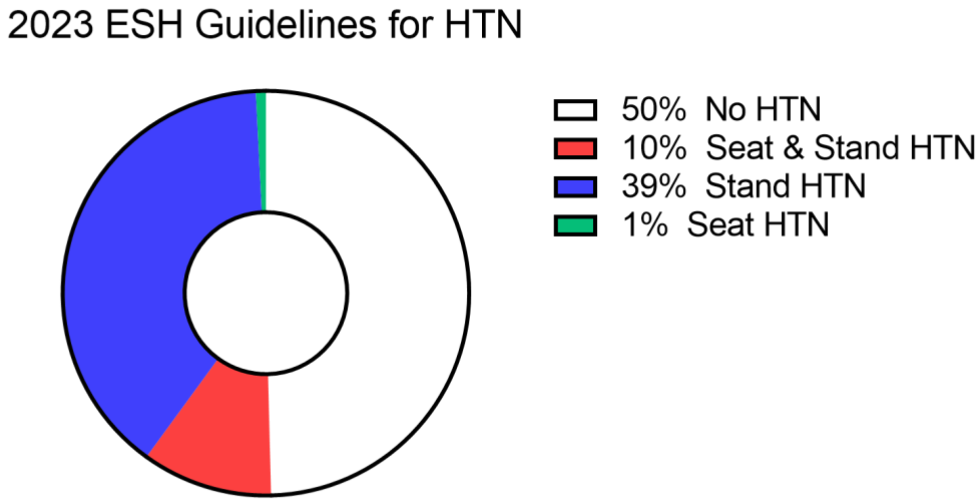

Supplement: Supplementary file 1 — Supplementary Information. [file 41598_2023_42297_MOESM1_ESM.pdf]
